# Measles resurgence in Italy: analysis of temporal patterns of viral transmission, surveillance performance, and vaccination coverage at national and regional levels, October 2023 to March 2025

**DOI:** 10.2807/1560-7917.ES.2026.31.45.2600090

**Published:** 2026-11-12

**Authors:** Valentina Baccolini, Antonio Sciurti, Erika Renzi, Azzurra Massimi, Giulia Bonanno, Serena Brunone, Benedetta Dellisanti, Marianna Riccio, Romina Pausilli, Chiara Tommasino, Martina Del Manso, Antonino Bella, Antonietta Filia, Silvia Gioacchini, Paola Bucci, Raoul Fioravanti, Emilio D’Ugo, Anna Teresa Palamara, Paolo Villari, Fabio Magurano, Carolina Marzuillo

**Affiliations:** 1 Department of Public Health and Infectious Diseases, Sapienza University of Rome, Rome, Italy; 2 National Verification Committee for Measles and Rubella Elimination, Italy; 3 Department of Life Sciences, Health, and Health Professions, Link Campus University, Rome, Italy; 4 Department of Infectious Diseases, Istituto Superiore Di Sanità, Rome, Italy

**Keywords:** Measles, measles vaccine, transmission, epidemiological surveillance, molecular surveillance

## Abstract

**BACKGROUND:**

Following substantial progress towards measles elimination after the COVID-19 pandemic, Italy experienced a marked increase in measles cases in 2024.

**AIM:**

Our aim was to describe measles epidemiology in Italy, October 2023–March 2025, and assess vaccination coverage, surveillance performance at national and regional levels, and temporal patterns of transmission.

**METHODS:**

Numbers of measles cases and outbreaks were reviewed. Vaccination coverage and surveillance indicators were described at national and regional levels. Measles variants were classified by genotype and Distinct Sequence Identifier (DSId). Temporal transmission patterns of measles variants were assessed nationally and regionally to identify sustained transmission chains (≥ 12 consecutive months).

**RESULTS:**

In total, 1,311 measles cases were reported, with 178 outbreaks. Cases’ median age was 30.9 years (IQR: 15.6–39.8); 601 were female (45.8%) and 710 male (54.2%). Only 3.1% (38/1,230) were fully vaccinated. In 2024, the first-dose vaccination coverage increased to 94.77% (95% CI: 94.70–94.84%) nationally, while second-dose coverage declined to 84.45% (95% CI: 84.35–84.55%). Among all cases, 12.1% (n = 158) were imported/importation-related, 64.1% (n = 841) were of unknown origin and 52.8% had DSId data available (n = 692), with high regional heterogeneity. Twenty-five measles variants were identified. One variant (D8–5963) showed sustained national transmission for over 12 months, meeting criteria for re-established endemic transmission, although no single region showed uninterrupted ≥ 12-month circulation.

**CONCLUSION:**

Measles resurgence in Italy was marked by persistent immunity gaps, heterogeneous surveillance performance, and sustained national circulation of a D8 variant, indicating loss of near-elimination status. Strengthening vaccination strategies and enhancing molecular surveillance is urgently needed to restore progress towards measles elimination.

**Key public health message**

**What did you want to address in this study and why?**

Measles can be preventable by vaccination, which is provided in two doses in Italy. After 2 years of progress towards elimination, measles case numbers in Italy increased dramatically in 2024. We wanted to understand, who was affected, how vaccination and surveillance systems were performing and how the measles virus spread over time. This is important because measles virus can quickly re-establish its circulation if protection in the population weakens.

**What have we learnt from this study?**

We found that measles mainly affected unvaccinated people, especially adolescents and young adults, reflecting long-standing vaccination gaps. Nationwide, the proportion of people vaccinated with two vaccine doses was below internationally recommended thresholds. We also saw regional differences in how well cases were detected and investigated. One measles virus strain circulated across the country for over a year, indicating re-established endemic transmission in Italy.

**What are the implications of your findings for public health?**

To halt measles resurgence in Italy, it is urgent to boost vaccination, particularly second doses and to conduct catch-up campaigns for adolescents and young adults. Health authorities must also standardise and improve surveillance and laboratory capacity across all regions to better detect and trace outbreaks. Coordinated national and regional effort is essential to keep progress towards measles elimination.

## Introduction

Measles is highly contagious and once transmission of measles virus is controlled, infections can rapidly re-occur when pockets of susceptible individuals accumulate [1]. This mainly explains why, while measles elimination is considered biologically and technically feasible [2], achieving it and then sustaining it is particularly challenging [3]. In 2014, to support national planning and efforts towards elimination, Member States in the World Health Organization (WHO) European Region jointly developed a concrete strategy, which outlines goals, priority actions and monitoring mechanisms [4]. In parallel, WHO established a framework for verifying measles elimination, providing definitions of key terms, core surveillance indicators and a description of the verification process [5]. Within this framework, each country is expected to produce an annual report on its elimination status (known as annual status update, ASU), containing standardised documentation based on the systematic collection and analysis of essential data. This evidence is compiled, analysed and validated by the National Verification Committee (NVC) and submitted annually to the Regional Verification Commission (RVC) for Measles and Rubella Elimination, that conducts an independent review process to further validate each Member State’ ASU [6].

After reviewing the Member States’ ASUs, the European RVC concluded that, by the end of 2023, 33 Member States had interrupted endemic measles transmission for ≥ 36 months and a further seven for ≥ 24 months. The RVC nevertheless emphasised that verification of measles elimination (i.e. interruption for at least 36 months) depends on continued immunisation, high-quality surveillance, and good laboratory performance [7]. Reports from RVC also emphasise that elimination can be reversed if immunity gaps permit prolonged transmission to re-emerge [7]. This point is particularly relevant because, while measles circulation was reported as having sharply dropped during the COVID-19 pandemic [8], likely due to a combination of reduced transmission and under-detection or under-reporting [9], a marked measles resurgence was thereafter observed in many countries of the Region, culminating in 2024 with very high reported case counts [10,11]. To promptly limit the virus circulation and prevent the re-emergence of sustained transmission, closing immunity gaps by increasing two-dose coverage, coupled with effective case-based surveillance and rapid outbreak response, is generally recommended [11].

In the last months of 2023, Italy experienced a surge in measles virus circulation, following a 2-year period in which endemic transmission had been interrupted [7,11]. In this context, integrating outbreak epidemiology with laboratory and molecular evidence is central to distinguish imported cases with limited onward spread from patterns compatible with prolonged transmission and endemic re-emergence [12]. Therefore, to provide actionable evidence for optimising risk-based public health actions, comprising tailored immunisation activities and control measures, the objective of this study was to describe measles epidemiology in Italy from October 2023 to March 2025. This included assessing the epidemiological situation regarding this disease, as well as the measles vaccine coverage and the national and regional performance of surveillance systems. Moreover, another aim was to understand temporal patterns of measles circulation at the national and regional levels, by combining available epidemiological information with molecular virological data.

## Methods

### Data sources

#### Measles case data

We used data on measles cases reported to the Italian National Integrated Measles and Rubella Surveillance System from October 2023 to March 2025. The surveillance system, established in 2013 and updated in 2018 [13], involves all 21 Italian Regions and Autonomous Provinces (hereafter referred to as regions), and is coordinated by the Department of Infectious Diseases of the Italian National Institute of Health (Istituto Superiore di Sanità, ISS).

Briefly, physicians are required to notify suspected measles cases to Local Health Authorities (LHA) within 12 hours of clinical suspicion. The LHAs carry out epidemiological investigations, collect information on cases, and notify the Regional Health Authorities (RHA), that subsequently report case information to the Department of Infectious Diseases of the ISS.

The LHAs also collect clinical specimens from suspected cases and forward them to Regional Reference Laboratories (RRLs), where serological or molecular testing is performed to confirm cases. Cases that test positive by molecular methods are sequenced, and the resulting sequence data are transmitted to the National Reference Laboratory (NRL) at the ISS in Rome. In Regions without an RRL, specimens are sent directly to the NRL. Currently, 15 RRLs operate across 14 Regions, constituting the Sub-national Reference Laboratories Network for measles and rubella (MoRoNET), coordinated by the NRL [14,15].

For this study, the following epidemiological information about measles cases was used: date of birth, sex (collected as binary variable: female/male), Region of notification, date of symptom onset (rash, fever, other clinical manifestations), emergency department visit and hospitalisation, availability of clinical samples, vaccination status and number of vaccine doses received, travel history, and contacts with other confirmed cases.

Data from phylogenetic analysis of sequences from the C-terminal region of the measles virus nucleoprotein gene (N-450), performed by the NRL, enables the identification of measles virus genotypes and variants, with the corresponding Distinct Sequence Identifier (DSId) obtained from the WHO Global Measles Nt Sequence Database (MeaNS2) [16,17].

#### Measles vaccination coverage data

In Italy, the monovalent measles vaccine was introduced in 1976 and was replaced by the combined measles-mumps-rubella (MMR) vaccine in the early 1990s [18]. Initially offered as a single dose, the schedule transitioned in 2003 to a routine two-dose scheme alongside the adoption of the first national measles elimination plan [18]. Currently, the MMR vaccine is recommended at 12 months of age (first dose) and at 5 years of age (second dose). Since 2017, MMR vaccination has been included among the mandatory vaccinations for children under 16 years of age for admission to state schools [19,20].

For this study, measles vaccination coverage in Italy from 2022 to 2024 for the first and second doses, corresponding to children aged 24 months and 6 years, respectively, was obtained from the Italian Ministry of Health [21]. The Directorate General for Health Emergencies of the Ministry of Health annually monitors measles vaccination coverage by requesting aggregated data on vaccine administrations from RHAs for specific birth cohorts (i.e. children aged 2 and 6 years), up to 31 December of the year preceding the request [21].

#### Case definitions

Measles cases were classified as possible, probable, or laboratory-confirmed according to the 2018 European Commission case definition [22]. In addition, they were classified as outbreak-related or sporadic: an outbreak was defined as two or more cases, including at least one laboratory-confirmed case, that were temporally related (with symptom onset occurring 7 to 18 days apart) and linked according to epidemiological (e.g. geographically related) and/or virological evidence (i.e. cases with the same DSId) [12]. Cases belonging to the same outbreak were assigned a common outbreak identification number. Conversely, sporadic cases were defined as cases for which no outbreak could be identified, based on the absence of both epidemiological and virological evidence.

Moreover, based on travel history [12], cases were also classified as imported (i.e. if the person had travelled outside Italy 7–23 days before symptom onset and epidemiological or virological evidence supported infection abroad), import-related (i.e. cases related to outbreaks secondary to an imported case as supported by epidemiological and/or virological evidence), or locally-acquired (i.e. cases that were neither imported nor import-related).

Sporadic, locally-acquired cases and the primary cases of locally- acquired outbreaks were defined as cases of unknown origin [12].

Based on the availability of genotype and DSId information, cases were classified according to identified viral genotype (i.e. B3, D8 or not available) and DSId (e.g. 5963). Cases without a molecularly determined DSId, but which were part of an outbreak with at least one case having an identified DSId, were assigned the same DSId as cases of this outbreak.

### Data analysis

#### Measles epidemiology, vaccination coverage and surveillance performance

We reported the following information for measles cases, both at the national and regional level: number of measles cases; number of measles outbreaks and minimum and maximum number of cases per outbreak; incidence of measles per million inhabitants; median case age and interquartile range (IQR); number and proportion of female and male cases; number and proportion of hospitalised cases; number and proportion of cases vaccinated with two doses among cases with known vaccination status. The proportion of hospitalised cases was compared between individuals who received at least one vaccine dose and unvaccinated individuals using a chi-squared test. A two-sided p value < 0.05 was considered statistically significant.

In addition, as indicators of the surveillance system performance, for each Region we calculated the following proportions with their 95% confidence intervals (CIs) and compared them with the respective national benchmark: proportions of outbreak-related cases; imported or import-related cases; cases with unknown origin; and cases with available DSId.

Measles vaccination coverage for the first and second doses in 2022, 2023, and 2024 was reported for Italy and all Regions, together with the corresponding 95% CIs, and contrasted with the WHO-recommended 95% vaccination coverage target [23].

#### Temporal patterns of measles transmission

Cases reported in the 18-month study period were grouped by identified measles variant (genotype and DSId, when available) and month of symptom onset. As the WHO defines the re-establishment of endemic measles transmission as “a situation in which epidemiological and laboratory evidence indicates the presence of a chain of transmission of a virus variant that continues uninterrupted for a period of 12 months or more in a defined geographical area where disease was previously eliminated” [12], a transmission chain of a given measles variant was considered sustained if at least one measles case with that genotype-DSId was reported for 12 consecutive months or longer during the study period. A single-month interruption – i.e. zero cases reported for a given variant in a specific month – was considered sufficient to break a transmission chain. Remaining cases lacking either genotype or DSId information were not considered as part of the same transmission chain. Analyses were conducted at both national and regional levels, with sensitivity analyses restricted to locally acquired cases only.

All analyses were conducted using anonymised data with R Statistical Software [24].

## Results

### Measles epidemiology, vaccination coverage and surveillance performance

Between October 2023 and March 2025, a total of 1,311 measles cases were reported in Italy, corresponding to an overall incidence of 19.6 cases per million inhabitants (Table). Overall, 178 measles outbreaks were identified, including four outbreaks involving multiple Regions, with a maximum of 19 cases per outbreak. Five Regions accounted for ca 70% of cases: the highest number of notifications was reported in Lazio (n = 227 cases, n = 24 outbreaks), followed by Sicilia (n = 223 cases, n = 30 outbreaks), Emilia-Romagna (n = 173 cases, n = 30 outbreaks), Lombardia (n = 153 cases, n = 21 outbreaks) and Campania (n = 130 cases, n = 18 outbreaks). The median age of cases was 30.9 years (IQR: 15.6–39.8), ranging between 17.2 and 38 years across Regions, with younger ages observed in Basilicata and Sicilia (around 17 years). Female and male individuals accounted for 45.8% (n = 601) and 54.2% (n = 710) of cases, respectively. Hospitalisation was required for half of the cases (50.1%, n = 657), and no deaths were observed. Only 3.1% of cases with known vaccination status had received two vaccine doses (38/1,230), and nine Regions reported no fully vaccinated cases, while in the remaining 10 Regions with at least one vaccinated case, the proportion of fully vaccinated cases ranged between 1.7% (Campania, 2/121) and 8.5% (Toscana, 5/59). Measles cases who received two doses and those vaccinated with one dose did not significantly differ by age (two doses: median: 27 years, IQR: 17.3–29.8; one dose: median: 25 years, IQR: 4.6–33.4; p = 0.322), sex (two doses: 11/38 male, and 27/38 female; one dose:  34/84 (40.5%) male, 50/84 (59.5%) female; p = 0.308). Among subjects with known vaccination status, those who received at least one vaccine dose had a lower proportion of hospitalisation (29.5%, 36/122) compared with unvaccinated individuals (51.4%, 569/1,108) (p < 0.001).

**Table:** Characteristics of measles cases in Italy and Italian Regions, October 2023–March 2025 (n = 1,311)

Region or Autonomous Province	Number of cases	Number of outbreaks	Min.–max. number of outbreak cases	Incidence: number of cases/million inhabitants^a^	Median case age in years (IQR)	Female cases		Male cases		Hospitalised cases		Number of two-dose vaccinated cases among cases with known vaccination status (%)^b^		
						Number	%	Number	%	Number	%			
Piemonte	10	2	2–2	1.2	22.3 (8.5–36.3)	3	30.0	7	70	3	30.0	0	10	(0.0)
Valle d’Aosta/Vallée d’Aoste	0	0	NA	0	NA	NA	NA	NA	NA	NA	NA	NA	NA	NA
Lombardia	153	21	2–6	12.7	35.1 (23.9–41.8)	63	41.2	90	58.8	78	51.0	5	142	(3.5)
Bolzano/Bozen	41	9	2–8	74.5	27.4 (16.4–39.7)	17	41.5	24	58.5	14	34.1	0	40	(0.0)
Trento	10	2	2–3	16.5	38.0 (27.6–48.7)	4	40.0	6	60	3	30.0	0	9	(0.0)
Veneto	46	5	2–5	5.2	36.6 (24.3–44.6)	20	43.5	26	56.5	13	28.3	2	45	(4.4)
Friuli-Venezia Giulia	17	4	2–3	7.5	27.7 (24.2–44.4)	9	52.9	8	47.1	9	52.9	0	17	(0.0)
Liguria	60	5	2–13	37.8	34.1 (27.7–45.8)	19	31.7	41	68.3	23	38.3	2	56	(3.6)
Emilia-Romagna	173	30	2–7	33.7	34.1 (20.1–44.1)	73	42.2	100	57.8	57	32.9	9	163	(5.5)
Toscana	63	4	2–17	14.7	33.9 (23.2–46.6)	42	66.7	21	33.3	24	38.1	5	59	(8.5)
Umbria	0	0	NA	0	NA	NA	NA	NA	NA	NA	NA	NA	NA	NA
Marche	34	4	2–5	15.5	29.5 (12.3–41.6)	15	44.1	19	55.9	17	50.0	2	29	(6.9)
Lazio	227	24	2–19	35.5	31.9 (24.8–41.7)	112	49.3	115	50.7	133	58.6	6	213	(2.8)
Abruzzo	48	5	2–3	34.6	36.3 (30.6–41.8)	19	39.6	29	60.4	34	70.8	1	43	(2.3)
Molise	1	0	NA	3.5	22.7 (22.7–22.7)	1	100.0	0	0	1	100.0	0	1	(0.0)
Campania	130	18	2–3	20.4	27.1 (9.4–33.7)	62	47.7	68	52.3	86	66.2	2	121	(1.7)
Puglia	48	6	2–15	12.1	21.9 (4.3–36.6)	25	52.1	23	47.9	31	64.6	0	45	(0.0)
Basilicata	4	1	3–3	5.6	17.2 (15.5–18.6)	3	75.0	1	25	4	100.0	0	3	(0.0)
Calabria	10	2	2–2	4.9	29.8 (28–33.9)	4	40.0	6	60	7	70.0	0	10	(0.0)
Sicilia	223	30	2–6	46.3	17.3 (4.1–31.5)	102	45.7	121	54.3	114	51.1	4	212	(1.9)
Sardegna	13	2	4–5	7	35.2 (30.9–37.6)	8	61.5	5	38.5	6	46.2	0	12	(0.0)
**Italy**	**1,311**	**178^c^**	**2–19**	**19.6**	**30.9 (15.6–39.8)**	**601**	**45.8**	**710**	**54.2**	**657**	**50.1**	**38**	**1,230**	**(3.1)**

IQR: Interquartile range; NA: not applicable; max: maximum; min: minimum.

^a^ A total of 158 cases was excluded from the incidence numerator because they were imported or import-related; the denominator was the average population residing in Italy/Regions up to 1 January in 2023, 2024 and 2025 [51].

^b^ A total of 81 cases with unknown vaccination status were excluded from the proportion denominator.

^c^ Four outbreaks involved multiple Regions.

Regarding measles vaccination coverage, first-dose uptake in Italy remained below 95% between 2022 and 2024, but showed a slight but steady increase, rising from 94.40% (95% CI: 94.33–94.47%) to 94.77% (95% CI: 94.70–94.84%) (Figure 1, panel A). In the last year, seven Regions reported coverages below 95% (Bolzano, Liguria, Marche, Campania, Puglia, Calabria, Sardegna), while in Sicilia coverage was below 90% (Figure 1, panel A). However, compared with 2023, vaccination coverage in 2024 declined by 0.1–1% in six Regions (Valle d’Aosta, Toscana, Trento, Umbria, Marche, Lazio), and in three Regions by more than 2% (Sicilia, Friuli-Venezia Giulia and Campania). Second dose coverage, instead, showed a small but progressive decline at the national level, decreasing from 85.06% (95% CI: 84.96–85.16) in 2022 to 84.45% (95% CI: 84.35–84.55%) in 2024. None of the Regions reached the 95% WHO target for the second dose, and nine Regions reached coverage above 90% (Piemonte, Valle D’Aosta, Lombardia, Trento, Veneto, Friuli-Venezia Giulia, Emilia-Romagna, Toscana, Umbria) (Figure 1, panel B). Measles vaccination coverage and its associated 95% CIs by Region are reported in Supplementary Table S1.

**Figure 1:**
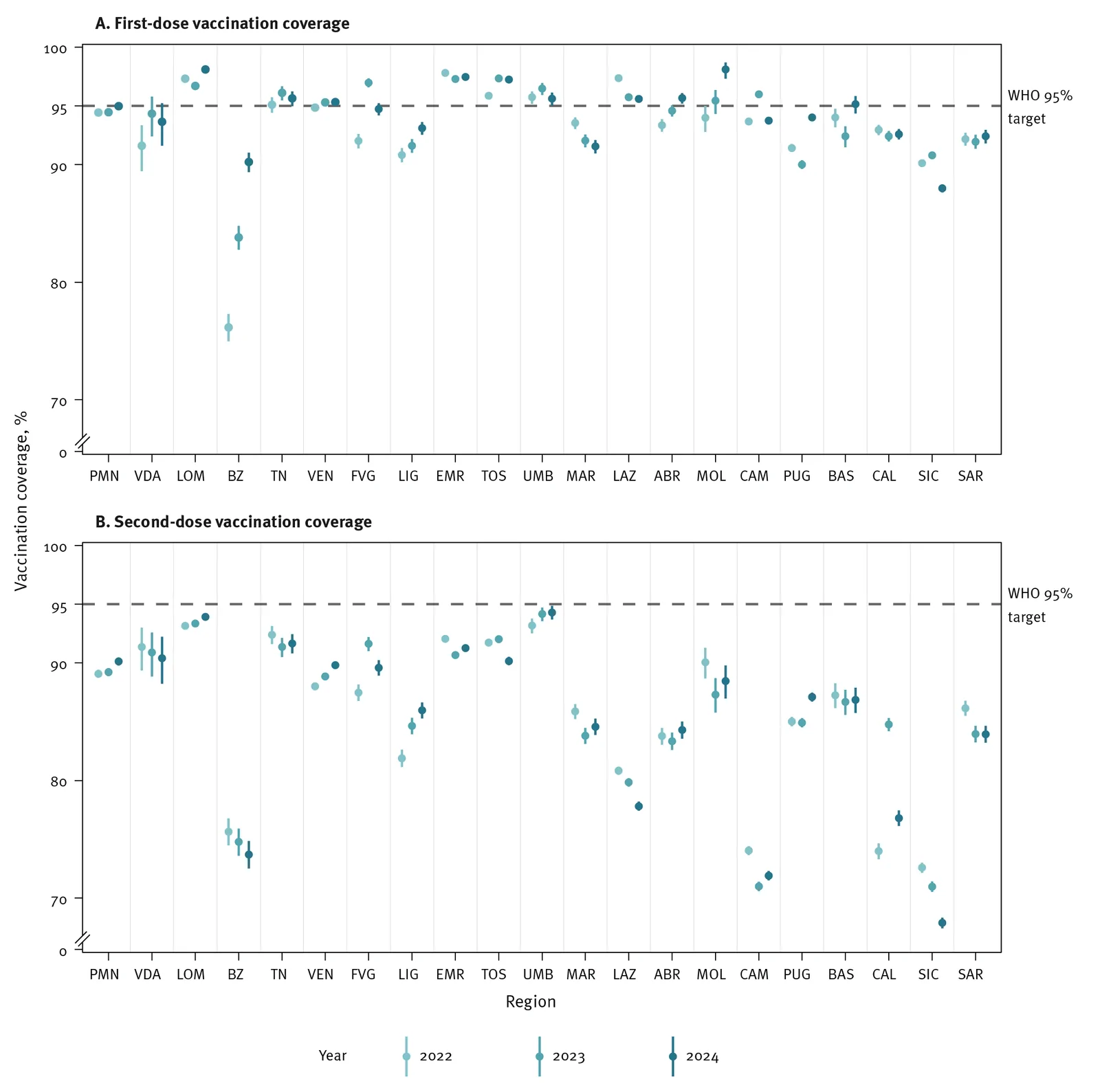
Measles vaccination coverage and 95% confidence intervals for the (A) first and (B) second dose in Italian Regions and Autonomous Provinces, in 2022, 2023 and 2024

ABR: Abruzzo; BAS: Basilicata; BZ: Bolzano/Bozen; CAL: Calabria; CAM: Campania; EMR: Emilia-Romagna; FVG: Friuli-Venezia Giulia; LAZ: Lazio; LIG: Liguria; LOM: Lombardia; MAR: Marche; MOL: Molise PMN: Piemonte; PUG: Puglia; SAR: Sardegna; SIC: Sicilia; TN: Trento; TOS: Toscana; UMB: Umbria; VDA: Valle d’Aosta/Vallée d’Aoste; VEN: Veneto; WHO: World Health Organization.

Each % coverage is presented as a dot with whiskers representing the respective 95% confidence interval. Vaccination coverage 95% confidence intervals were computed based on available vaccination coverage point-estimates [21] and population aged 2 and 6 years residing in Italy/Italian Regions up to 1 January in 2022, 2023 and 2024 [51] according to the Wilson method [52].

Nationally, 41.7% (n = 547) of measles cases reported in Italy were outbreak-related (Figure 2, panel A). Compared with the national value, Bolzano, Friuli-Venezia Giulia, and Puglia reported higher proportions of outbreak-related cases, between 63.4% (n = 26) and 77.1% (n = 37), while Campania reported a lower proportion (28.5%, 95% CI: 21.1–37.2%, n = 37). Imported or importation-related cases accounted for 12.1% (n = 158) nationally (Figure 2, panel B). This proportion was significantly higher in Piemonte, Veneto, Friuli-Venezia Giulia, and Marche, where it ranged between 32.4% (n = 11) and 50.0% (n = 5), and was significantly lower in Sicilia (0.4%, 95% CI: 0.0–2.9%, n = 1). Cases with unknown origin represented 64.1% (n = 841) of cases in Italy (Figure 2, panel C), with higher proportions of unknown origin-cases observed in Campania (76.2%, 95% CI: 67.7–83.0%, n = 99) and Sicilia (77.1%, 95% CI: 70.9–82.4%, n = 172). Finally, clinical samples were available for 75.5% cases (n = 993), and unavailable for 24.3% (n = 318).

**Figure 2:**
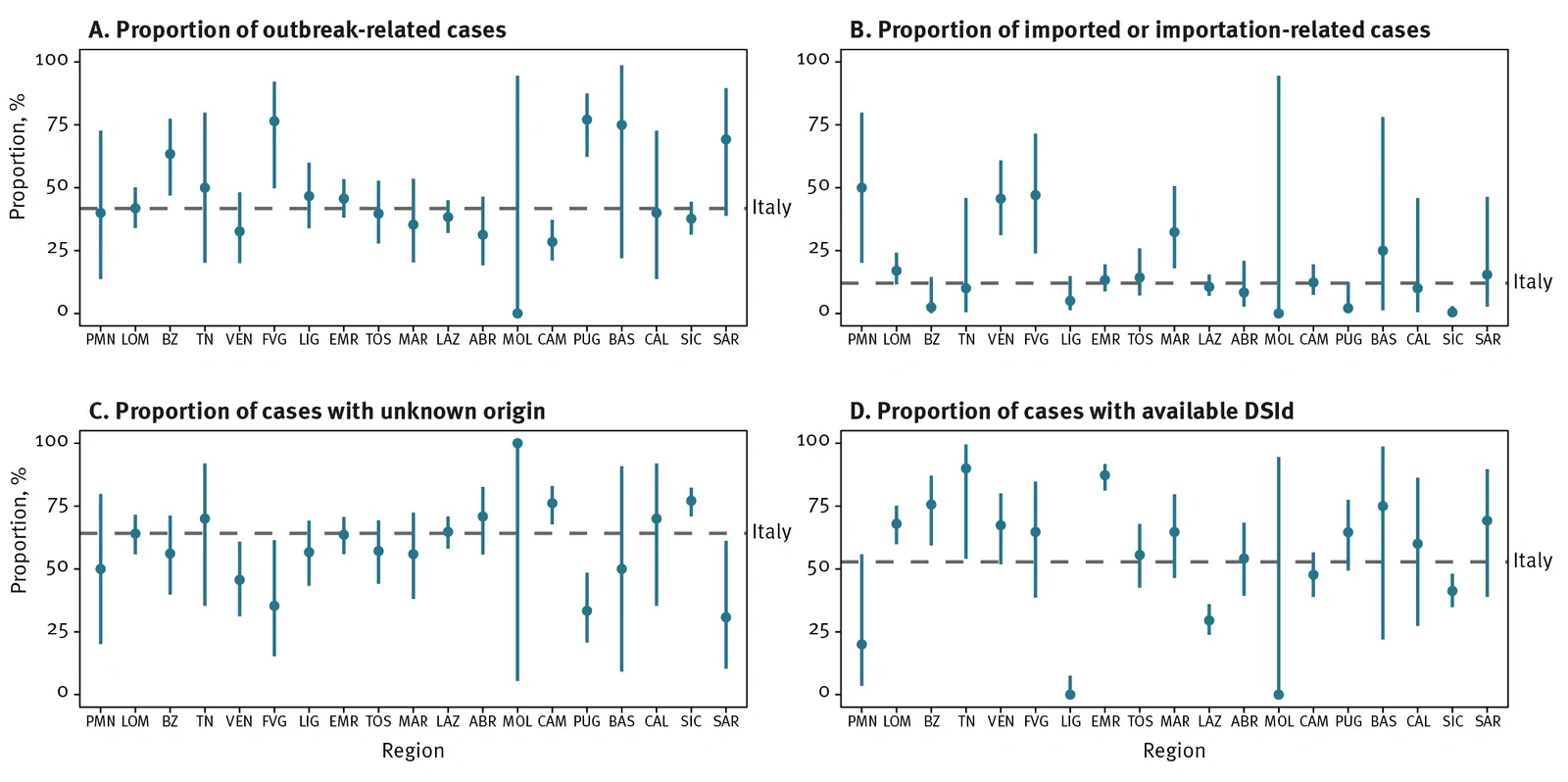
Proportion and 95% CIs of (A) outbreak-related cases, (B) imported or importation-related cases, (C) cases with unknown origin and (D) cases with DSId available in Italy and Italian Regions and Autonomous Provinces^a^, October 2023–March 2025 (n = 1,311)

95% CI: 95% confidence interval; DSId: Distinct Sequence Identifier.

^a^ Autonomous Provinces and Regions include: ABR: Abruzzo; BAS: Basilicata; BZ: Bolzano/Bozen; CAL: Calabria; CAM: Campania; EMR: Emilia-Romagna; FVG: Friuli-Venezia Giulia; LAZ: Lazio; LIG: Liguria; LOM: Lombardia; MAR: Marche; MOL: Molise; PMN: Piemonte; PUG: Puglia; SAR: Sardegna; SIC: Sicilia; TN: Trento; TOS: Toscana; VEN: Veneto. Valle d’Aosta and Umbria are not represented on the figure because no measles cases were reported there.

Each % coverage is presented as a dot with whiskers representing the respective 95% confidence interval. Proportions’ 95% CIs were computed according to the Wilson method [52].

Testing by PCR was performed for all cases with available clinical samples, but DSId was characterised in only 56.5% cases (561/993). Additionally, for this study 131 cases with unavailable sample or without DSId characterisation were assigned a common DSId as they were part of outbreaks with at least one case with an identified DSId. Thus, DSId information was available for 692 cases in Italy (52.8%) (Figure 2, panel D). Lombardia, Bolzano, Trento, and Emilia-Romagna showed higher proportions of cases with DSId information, from 68.0% (n = 104) to 90.0% (n = 9), while Liguria, Lazio, and Sicilia reported lower proportions, from 0.0% (n = 0) to 41.3% (n = 92). The corresponding proportions and 95% CIs in each Region are shown in Supplementary Table S2.

### Temporal patterns of measles transmission

Over the 18-month period, 42.8% (n = 561) of cases had DSId determined from clinical samples, 18.1% (n = 237) had only genotype data available, including 206 and 31 with D8 and B3 genotype, respectively, and 39.1% of cases (n = 513) had no genotype data. Of 750 cases with undetermined DSId, 131 were part of outbreaks for which at least one case had DSId, so they were assigned this DSId. In this way, we were able to identify a DSId for 692 cases overall. Among the total 692 cases with DSId, 25 measles variants with unique DSId were detected across Italy. The most frequently isolated variant was D8–8491 (28.0%, 194/692), followed by D8–5963 (15.8%, 109/692), B3–6418 (11.7%, 81/692), D8–8845 (9.2%, 64/692), D8–8350 (8.2%, 57/692) and D8–8248 (7.7%, 53/692). All other variants accounted for less than 5% each. Across Regions, Lombardia showed the highest number of identified variants (13 variants, n = 104 cases), followed by Emilia-Romagna and Toscana (nine variants each, n = 151 and n = 35 cases, respectively), Veneto (eight variants, n = 31 cases), Lazio (seven variants, n = 67 cases), Sicilia, Campania and Marche (five variants each, n = 92, n = 62, n = 22 cases, respectively), while in other Regions less than five variants were identified (Figure 3).

**Figure 3:**
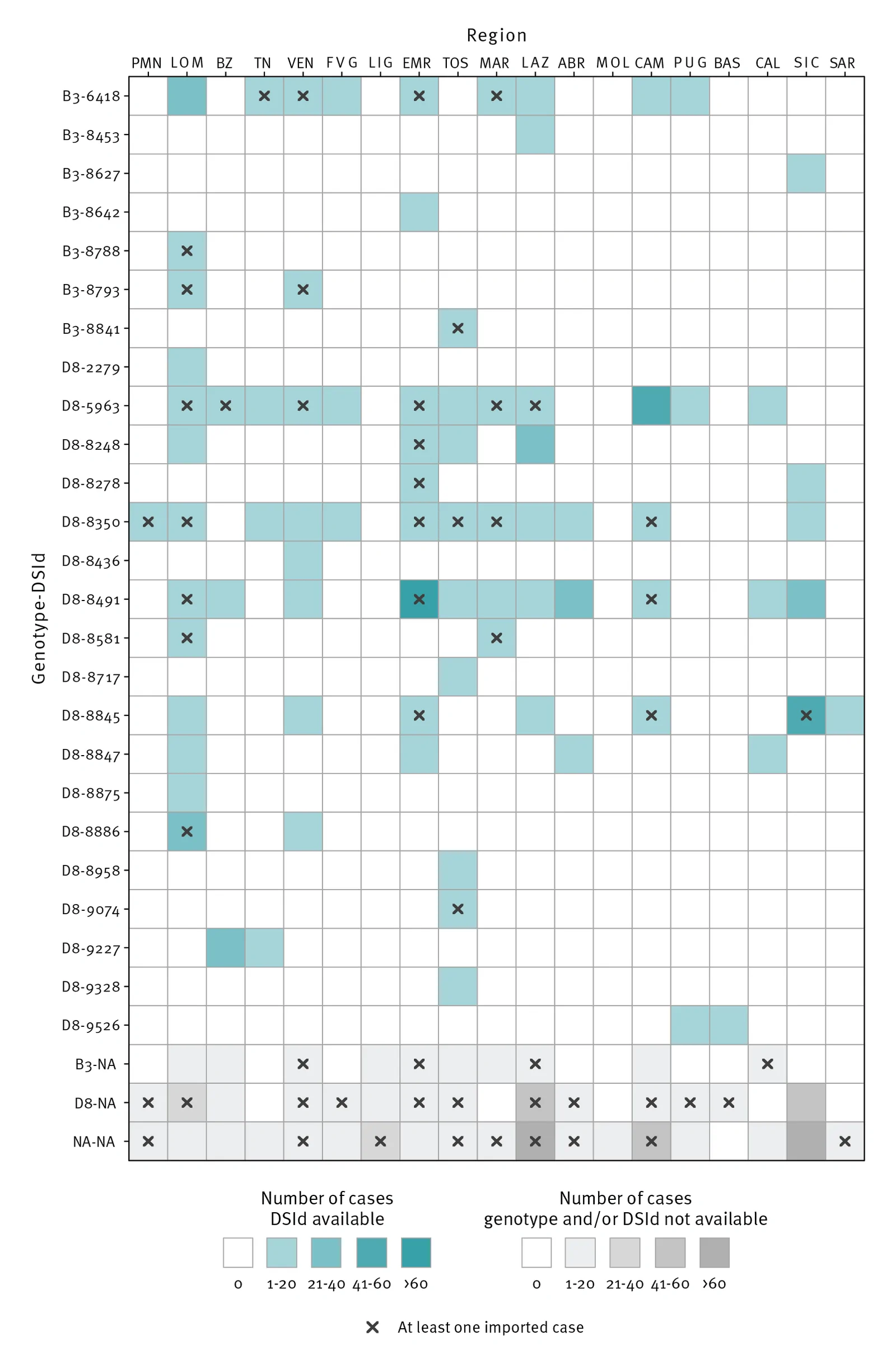
Measles viruses identified in Italian Regions, October 2023–March 2025 (n = 1,311)

NA: not available.

ABR: Abruzzo; BAS: Basilicata; BZ: Bolzano/Bozen; CAL: Calabria; CAM: Campania; EMR: Emilia-Romagna; FVG: Friuli-Venezia Giulia; LAZ: Lazio; LIG: Liguria; LOM: Lombardia; MAR: Marche; MOL: Molise; PMN: Piemonte; PUG: Puglia; SAR: Sardegna; SIC: Sicilia; TN: Trento; TOS: Toscana; VEN: Veneto.

Valle d’Aosta and Umbria are not represented in the figure because no measles cases were reported there. In the figure’s table, × denotes at least one imported case.

Time patterns of measles transmission between October 2023 and March 2025 in Italy are shown in Figure 4. At the national level, variants belonging to the B3 genotype showed limited transmission in 2024. The B3–6418 variant was first detected in January 2024 and was then identified intermittently across 2024 (April, June and a short episode in August–September), before reappearing again in early 2025 and being detected through March 2025. Other B3 variants were observed only in 2024, with limited spread and transmission periods of 2 months or less (often as single-month detections). In contrast, D8 variants showed longer transmission periods overall. The D8–5963 variant was the only one showing a sustained chain of transmission lasting ≥ 12 months, spanning from January 2024 to March 2025. Geographical patterns of transmission for the D8–5963 variant are shown in Supplementary Figure S1. Other D8 variants with prolonged circulation included D8–8491 (from November 2023 to July 2024), D8–8350 (from April 2024 to September 2024) and D8–8845, that was found from July to September 2024 and then again from November 2024 to March 2025. The remaining D8 variants were mainly detected in 2024 and generally for no longer than 3 months.

**Figure 4:**
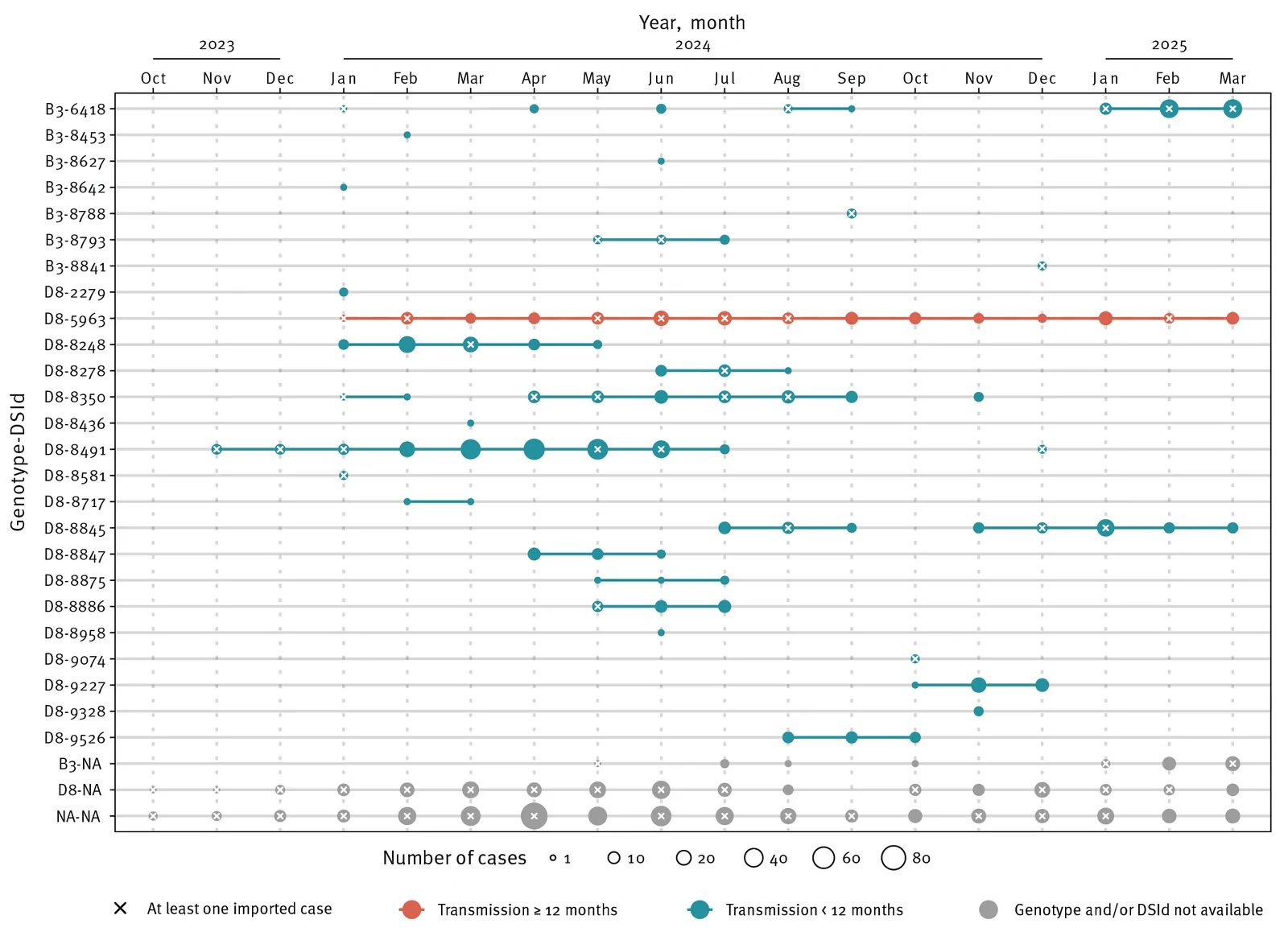
Measles identified variants in Italy, by month, October 2023–March 2025 (n = 1,311)

NA: not available.

The size of points (i.e. circles) is proportional to the number of measles cases in a month. Months with at least one new imported case are denoted by an ×.

At the regional level, none of the measles variants showed transmission lasting 12 months or longer. The D8–5963 variant, isolated in 12 Regions (n = 109), was first detected in Lazio in January 2024, where it showed generally limited circulation, but had a longer uninterrupted transmission in Campania from April 2024 to February 2025 (11 months), and a shorter sustained period in Puglia (5 months, from September 2024 to January 2025). The D8–8350 variant circulated across 12 Regions (n = 57) with moderate persistence, reaching up to 6 months of continued transmission in Lazio and Lombardia during 2024. D8–8491 was isolated in 11 Regions (n = 194), first appearing at the end of 2023 in Campania and showing prolonged transmission in Lombardia and Emilia-Romagna from January to July 2024, alongside shorter circulation in Sicilia, Marche and Lazio. The B3–6418 variant (nine Regions, n = 81) was initially detected sporadically in 2024 but later showed more consistent transmission in the first 3 months of 2025, observed simultaneously in several northern Regions. The D8–8845 (seven Regions, n = 64) variant displayed intermittent circulation, with two distinct periods of sustained transmission in Sicilia (from July to September 2024, and from December 2024 through March 2025) and a shorter period in Sardegna (from January through March 2025), while the D8–8248 (four Regions, n = 53) variant showed limited spread overall, except for a longer transmission period in Lazio during the first half of 2024. All remaining variants were detected in four Regions or fewer and were characterised by brief transmission chains lasting no more than 3 months. Regional patterns of measles transmission are presented for each identified variant in Supplementary Figure S2.

No meaningful difference was observed when the analysis was restricted to locally acquired cases only (data not shown).

## Discussion

Amid the global resurgence of measles following the COVID-19 pandemic [10], Italy experienced a sharp increase in cases after a prolonged period of interrupted transmission [7], ranking as the second most affected country in the European Union (EU) in 2024 [11]. This surge in measles cases was characterised by pronounced geographic heterogeneity, evidence of multiple importations, and sustained circulation of one D8 variant, occurring against a background of persistent immunity gaps, especially among young adults.

Transmission predominantly involved unvaccinated individuals aged 15–39 years, likely reflecting long-standing gaps in vaccination coverage. While the introduction of mandatory childhood vaccination in 2017 led to improved coverage in childhood cohorts, substantial susceptibility persists among adolescents and young adults [25,26]. In this context, the missed measles/MMR vaccination has been attributed mainly to failure to attend scheduled appointments and informed refusal, within a broader context of vaccine hesitancy driven by safety concerns and misinformation [27,28]. Available institutional data indeed show below-target 2024 coverage estimates for several other recommended adolescent vaccines, including polio and diphtheria boosters, as well as HPV vaccination [29]. Several other European countries in 2024 [11], including France, Spain, and Poland, reported a substantial proportion of measles cases among young adults, whereas in Romania, where childhood vaccination coverage has declined markedly, cases were occurring predominantly among children [30]. This supports the idea that the age distribution of measles cases reflects differences in countries’ long-term vaccination uptake, potentially influenced by variations in national vaccination policies (e.g. mandatory vaccination) [31]. In addition, despite some variability throughout the country, first-dose coverage appears to have returned to pre-pandemic levels, whereas second-dose coverage has remained well below the 95% target and has continued to show pronounced and persistent regional heterogeneity, with generally lower values in southern Regions [32]. This discrepancy between high first-dose and suboptimal second-dose coverage is likely multifactorial. It has been partly attributed to the asymmetric enforcement of school-entry vaccination requirements, which are more stringent for preschool attendance than for entry into primary school [33], while other reported contributors include parental forgetfulness, potentially exacerbated by the long interval between doses, and residual complacency regarding the need to complete the two-dose schedule [34,35]. Nevertheless, this geographic pattern is particularly relevant considering the concentration of cases that was observed, a finding that suggests clustered susceptibility of individuals, likely resulting from historically uneven vaccination coverage across Regions [36]. It is further concerning in light of the recent declines in coverage observed in some areas (e.g. Sicilia and Campania), possibly reflecting reduced risk awareness and the absence of a coordinated national strategy. For these reasons, there is a need for strong commitment at both national and regional levels to close the second-dose gap and actively identify and immunise pockets of susceptible individuals through catch-up campaigns [11], particularly among adolescents and young adults and in those areas with lower coverage or higher incidence. Moreover, although the high proportion of cases requiring hospitalisation we documented may be biased by under-detection or under-reporting of milder cases [37], this finding underscores a substantial toll on the healthcare system [38], in terms of either morbidity and mortality of the patients or the economic impact on the institutions concerned. As nosocomial transmission seems to remain a relevant issue in Italy [39,40], and healthcare workers’ vaccination coverage – although increasing – remains suboptimal [40], national vaccination policies aimed at improving immunisation among healthcare workers are critical [39].

Regional differences were also observed in measles surveillance performance, as evidenced by marked variation in the accuracy and completeness of epidemiological and laboratory investigations across the country. Prior work has already argued that regional surveillance performance is partly associated with Italy’s healthcare decentralisation policies, which have exacerbated long-standing disparities in health service delivery across Regions, especially in health promotion and disease prevention activities [36]. Notwithstanding the inherent difficulty of tracing transmission chains for such a highly contagious virus as measles virus [41], the low proportion of outbreak-related cases probably indicates reduced capacity in conducting outbreak investigations. Relatedly, the rate of cases with unknown origin was generally much higher than the WHO’s 20% threshold, data that could suggest delayed detection, incomplete contact tracing or limited molecular resolution [12]. Indeed, full molecular characterisation was insufficiently available, with only a few Regions meeting the WHO target of viral genotyping and sequencing in ≥ 80% of sporadic cases [12]. In this regard, WHO has clearly stated that laboratory investigation has become increasingly important because both B3 and D8 genotypes have been detected since 2021, making genotype-level information alone insufficient to elucidate transmission patterns [16]. This was evident in our study, as incomplete DSId information limited the ability to fully reconstruct transmission chains in many Regions, and it likely inflated the number of cases classified as having an unknown origin and reduced sensitivity to detect sustained transmission. This limitation is highly relevant to distinguish endemic circulation from repeated importations with controlled transmission events, not only in formal terms, given the shift of Italy from a near-elimination status to the re-establishment of endemic transmission [7,42], but also in practical terms, as the public health actions required may differ substantially between these two scenarios at national and regional levels.

From a national perspective, evidence on time patterns of measles transmission suggested a scenario characterised by several importations [40] of different virus variants and prolonged transmission of a few of them; D8 variants predominated during 2024, in line with broader European trends [43], while B3 became increasingly prominent in 2025, consistent with recent local reports from Italy [44,45] and other EU countries [46,47]. Only one variant, D8–5963, met the WHO definition for re-established endemic transmission: this strain, commonly referred to as MVs/Patan.IND/16.19, was the second most frequently detected variant in the WHO European Region in 2024 [43] and showed extensive circulation also in Germany and Austria [46,47]. By contrast, our regional-level data did not clearly indicate sustained transmission lasting ≥ 12 months, even if we account for some degree of potential under-detection by requiring two consecutive months with zero cases (rather than one) to define interruption of a transmission chain. This finding points to a pattern of geographically shifting transmission that may reflect repeated importations, intermittent detection of the same variants within these areas, or both. As a result, no single Region showed evidence of stable endemic circulation, unlike the 2017 epidemic, during which sustained transmission chains were documented across almost all Italian Regions [48,49]. Notably, among the five Regions reporting the highest number of cases in 2024, Lombardia and Emilia-Romagna also had comparatively high sequencing coverage, that led to improved variant resolution and reduced uncertainty for cases lacking DSId. This higher level of completeness allowed a more accurate characterisation of measles transmission chains and did not support evidence of stable endemic transmission within these Regions. The decisive role of virological data in assessing endemic measles transmission has also been highlighted by the German NVC, which initially proposed interrupted transmission in 2024 [47] based on the identification of two partially overlapping clusters, neither of which persisted for more than 12 months. Given the minimal discrepancy between them, however, the RVC subsequently considered these clusters as belonging to a single variant, thereby re-establishing evidence of endemic transmission [42]. As for Italy, incomplete DSId coverage still limits definitive interpretation of measles transmission in many Regions and almost certainly leads to an underestimation of circulation in these areas. Importantly, this limitation does not undermine the national-level evidence of sustained circulation of the D8–5963 variant, that remains robust and indicate that the country has lost its near-elimination status and entered a phase of re-established endemic transmission [7], as recently confirmed by the RVC [42].

This study has several strengths. It draws on a national, legally mandated integrated surveillance system covering all Italian Regions, and it is among the first studies in Italy to integrate epidemiological investigation data with molecular characterisation and variant-specific temporal analyses, whereas previous reports have largely focused on local or regional outbreaks [44,45,50]. In addition, the comparison across Regions, in terms of both surveillance performance and vaccination coverage, provides insights into persistent subnational heterogeneity and is valuable for monitoring and harmonising progress towards elimination [36]. Several limitations should also be acknowledged. First, although we used the most comprehensive data available, genotype and DSId information was incomplete, which has probably biased estimates of variant diversity and transmission continuity at both national and regional levels. Specifically, because cases without DSId data could not be analysed, sustained transmission of individual variants has likely been underestimated, as cases reported in months without DSId information could have belonged to any of the identified variants. Moreover, albeit less likely, cases without DSId data could have also belonged to variants that were not detected in Italy. Second, these analyses rely on the accuracy and completeness of epidemiological investigations, which was suboptimal, particularly in some Regions. Moreover, underdiagnosis and under-reporting, especially of milder cases, may have affected the analyses, likely leading to an underestimation of the total case count or the duration of virus circulation. Finally, on a similar note, two Regions (i.e., Umbria and Valle d’Aosta) reported no cases during the study period; although surveillance was active in both, we cannot fully exclude under-ascertainment, which could have led us to underestimate the impact of the disease, as well. Therefore, future research should focus on identifying the drivers of regional differences in measles surveillance and control, including completeness of outbreak investigations and laboratory capacity, to align and support elimination efforts; identifying susceptible populations by age group and Region, to design more targeted catch-up interventions; and assessing the contribution of specific settings to adult transmission, such as healthcare facilities, schools, and workplaces, to further inform prevention strategies.

## Conclusion

Despite incomplete molecular data, which limited full reconstruction of measles transmission chains, our findings indicate that the 2024 resurgence in Italy was substantial, characterised by sustained national circulation of a D8 variant alongside persistent immunity gaps, particularly among young adults and in second-dose coverage among children. To interrupt re-established transmission and prevent further spread, coordinated national and regional strategies are urgently needed. Key priorities include closing coverage gaps, by strengthening two-dose routine childhood vaccination and identifying residual pockets of susceptibility among adolescents and young adults through targeted catch-up campaigns and enhancing surveillance and outbreak response by ensuring more accurate epidemiological and laboratory investigations.

## Data Availability

Aggregated data supporting reported results are available upon request. Individual-level data are unavailable due to privacy restrictions. Measles sequence data have been deposited in WHO's global Measles Virus Nucleotide Surveillance database (MeaNS) (https://who-gmrln.org/means2) and further information on the submitted sequences can be obtained from the NLR upon request.

## Supplementary Data

663000 Supplement
