# Supplementary material for: Measles resurgence in Italy: analysis of temporal patterns of viral transmission, surveillance performance, and vaccination coverage at national and regional levels, October 2023 to March 2025
Source: Euro Surveill. 2026 Nov 12;31(45):2600090. doi: 10.2807/1560-7917.ES.2026.31.45.2600090 (PMC13768767; doi:10.2807/1560-7917.ES.2026.31.45.2600090)
Supplement: Supplementary file 1 [file 26-00090-SCIURTI_Supplement.pdf]

This supplementary material is hosted by *Eurosurveillance* as supporting information alongside the article *Measles resurgence in Italy: analysis of temporal patterns of viral transmission, surveillance performance, and vaccination coverage at national and regional levels, October 2023-March 2025*, on behalf of the authors, who remain responsible for the accuracy and appropriateness of the content. The same standards for ethics, copyright, attributions and permissions as for the article apply. Supplements are not edited by *Eurosurveillance* and the journal is not responsible for the maintenance of any links or email addresses provided therein.

Supplementary Table S1. Measles vaccination coverage and 95% CIs for the first (A) and second dose (B) in Italy and Italian Regions, in 2022, 2023 and 2024.

Region	A. First-dose coverage, % (95% CI)			B. Second-dose coverage, % (95% CI)		
	**2022**	**2023**	**2024**	**2022**	**2023**	**2024**
**Italy**	**94.40 (94.33–94.47)**	**94.64 (94.57–94.71)**	**94.77 (94.70–94.84)**	**85.06 (84.96–85.16)**	**84.79 (84.69–84.89)**	**84.45 (84.35–84.55)**
Piemonte	94.44 (94.17–94.70)	94.47 (94.19–94.73)	94.98 (94.71–95.23)	89.08 (88.74–89.41)	89.22 (88.88–89.55)	90.13 (89.79–90.46)
Valle d'Aosta/Vallée d'Aoste	91.60 (89.44–93.36)	94.33 (92.41–95.80)	93.65 (91.61–95.23)	91.36 (89.37–93.01)	90.89 (88.85–92.60)	90.41 (88.24–92.22)
Lombardia	97.32 (97.20–97.43)	96.71 (96.58–96.84)	98.10 (98.00–98.20)	93.16 (92.99–93.33)	93.36 (93.19–93.53)	93.92 (93.75–94.08)
Bolzano/Bozen	76.15 (74.98–77.29)	83.81 (82.78–84.79)	90.22 (89.38–91.01)	75.64 (74.48–76.76)	74.77 (73.61–75.90)	73.69 (72.50–74.84)
Trento	95.11 (94.41–95.73)	96.11 (95.46–96.67)	95.65 (94.98–96.23)	92.40 (91.61–93.12)	91.35 (90.51–92.12)	91.66 (90.82–92.43)
Veneto	94.86 (94.62–95.09)	95.30 (95.07–95.52)	95.32 (95.09–95.54)	88.00 (87.68–88.32)	88.84 (88.52–89.15)	89.80 (89.49–90.10)
Friuli-Venezia Giulia	92.03 (91.39–92.62)	96.98 (96.56–97.35)	94.72 (94.18–95.21)	87.48 (86.77–88.16)	91.62 (91.02–92.18)	89.59 (88.92–90.22)
Liguria	90.82 (90.20–91.41)	91.61 (91.02–92.17)	93.10 (92.55–93.62)	81.90 (81.15–82.63)	84.64 (83.93–85.33)	85.97 (85.28–86.63)
Emilia-Romagna	97.81 (97.64–97.97)	97.28 (97.09–97.46)	97.47 (97.29–97.64)	92.05 (91.77–92.32)	90.66 (90.35–90.96)	91.27 (90.97–91.56)
Toscana	95.87 (95.61–96.12)	97.33 (97.11–97.53)	97.23 (97.01–97.44)	91.74 (91.41–92.06)	92.01 (91.68–92.33)	90.16 (89.80–90.51)
Umbria	95.73 (95.16–96.23)	96.47 (95.93–96.94)	95.61 (95.02–96.14)	93.17 (92.52–93.76)	94.16 (93.56–94.71)	94.30 (93.69–94.86)
Marche	93.54 (93.03–94.02)	92.04 (91.48–92.57)	91.56 (90.98–92.11)	85.87 (85.23–86.49)	83.80 (83.11–84.46)	84.58 (83.89–85.25)
Lazio	97.37 (97.21–97.52)	95.75 (95.54–95.95)	95.60 (95.39–95.80)	80.83 (80.48–81.18)	79.84 (79.48–80.20)	77.81 (77.43–78.19)
Abruzzo	93.36 (92.81–93.87)	94.59 (94.08–95.06)	95.66 (95.20–96.08)	83.77 (83.04–84.48)	83.35 (82.61–84.07)	84.29 (83.55–85.00)
Molise	93.99 (92.80–95.00)	95.45 (94.33–96.36)	98.11 (97.30–98.69)	90.06 (88.69–91.29)	87.30 (85.77–88.69)	88.46 (86.99–89.78)
Campania	93.68 (93.45–93.90)	95.98 (95.79–96.16)	93.74 (93.51–93.97)	74.03 (73.65–74.41)	70.99 (70.59–71.39)	71.89 (71.49–72.28)
Puglia	91.42 (91.08–91.75)	90.01 (89.64–90.37)	94.02 (93.73–94.30)	85.02 (84.62–85.41)	84.92 (84.52–85.31)	87.10 (86.72–87.47)
Basilicata	94.01 (93.18–94.75)	92.42 (91.48–93.26)	95.15 (94.35–95.84)	87.25 (86.17–88.25)	86.68 (85.58–87.71)	86.86 (85.76–87.89)
Calabria	92.96 (92.53–93.37)	92.43 (91.98–92.86)	92.60 (92.14–93.04)	73.98 (73.29–74.65)	84.76 (84.19–85.31)	76.80 (76.13–77.46)
Sicilia	90.14 (89.84–90.43)	90.79 (90.49–91.08)	87.98 (87.65–88.31)	72.58 (72.15–73.00)	70.97 (70.53–71.41)	67.88 (67.43–68.33)
Sardegna	92.18 (91.60–92.72)	91.96 (91.35–92.53)	92.41 (91.82–92.96)	86.15 (85.49–86.78)	83.95 (83.23–84.64)	83.94 (83.21–84.64)

95% CI: 95% Confidence Interval.

Vaccination coverage 95% CIs were computed based on available vaccination coverage point-estimates [21] and population aged two and six residing in Italy/Regions as of January 1st in 2022, 2023 and 2024 [24] according to the Wilson method [25].

Supplementary Table S2. Number, proportion and 95% CIs of outbreak-related cases (A), imported or importation-related cases (B), cases with unknown origin (C), cases with DSId available (D) in Italy and Italian Regions, October 2023-March 2025 (n = 1,311).

**Region**	**N. total cases**	**A. Outbreak-related cases**		**B. Imported or importation-related cases**		**C. Cases with unknown origin**		**D. Cases with DSId available**	
		**N.**	**% (95% CI)**	**N.**	**% (95% CI)**	**N.**	**% (95% CI)**	**N.**	**% (95% CI)**
**Italy**	**1,311**	**547**	**41.7**	**158**	**12.1**	**841**	**64.1**	**692**	**52.8**
Piemonte	10	4	40.0 (13.7–72.6)	5	50.0 (20.1–79.9)	5	50.0 (20.1–79.9)	2	20.0 (3.5–55.8)
Valle d'Aosta/Vallée d'Aoste	0	0	NA	0	NA	0	NA	0	NA
Lombardia	153	64	41.8 (34.0–50.1)	26	17.0 (11.6–24.1)	98	64.1 (55.9–71.5)	104	68.0 (59.9–75.1)
Bolzano/Bozen	41	26	63.4 (46.9–77.4)	1	2.4 (0.1–14.4)	23	56.1 (39.9–71.2)	31	75.6 (59.4–87.1)
Trento	10	5	50.0 (20.1–79.9)	1	10.0 (0.5–45.9)	7	70.0 (35.4–91.9)	9	90.0 (54.1–99.5)
Veneto	46	15	32.6 (20.0–48.1)	21	45.7 (31.2–60.8)	21	45.7 (31.2–60.8)	31	67.4 (51.9–80.0)
Friuli-Venezia Giulia	17	13	76.5 (49.8–92.2)	8	47.1 (23.9–71.5)	6	35.3 (15.3–61.4)	11	64.7 (38.6–84.7)
Liguria	60	28	46.7 (33.9–59.9)	3	5.0 (1.3–14.8)	34	56.7 (43.3–69.2)	0	0.0 (0.0–7.5)
Emilia-Romagna	173	79	45.7 (38.1–53.4)	23	13.3 (8.8–19.5)	110	63.6 (55.9–70.7)	151	87.3 (81.2–91.7)
Toscana	63	25	39.7 (27.8–52.8)	9	14.3 (7.1–25.9)	36	57.1 (44.1–69.3)	35	55.6 (42.6–67.9)
Umbria	0	0	NA	0	NA	0	NA	0	NA
Marche	34	12	35.3 (20.3–53.5)	11	32.4 (18.0–50.6)	19	55.9 (38.1–72.4)	22	64.7 (46.5–79.7)
Lazio	227	87	38.3 (32.0–45.0)	24	10.6 (7.0–15.5)	147	64.8 (58.1–70.9)	67	29.5 (23.8–36.0)
Abruzzo	48	15	31.2 (19.1–46.4)	4	8.3 (2.7–20.9)	34	70.8 (55.7–82.6)	26	54.2 (39.3–68.4)
Molise	1	0	0.0 (0.0–94.5)	0	0.0 (0.0–94.5)	1	100.0 (5.5–100.0)	0	0.0 (0.0–94.5)
Campania	130	37	28.5 (21.1–37.2)	16	12.3 (7.4–19.5)	99	76.2 (67.7–83.0)	62	47.7 (38.9–56.6)
Puglia	48	37	77.1 (62.3–87.5)	1	2.1 (0.1–12.5)	16	33.3 (20.8–48.5)	31	64.6 (49.4–77.4)
Basilicata	4	3	75.0 (21.9–98.7)	1	25.0 (1.3–78.1)	2	50.0 (9.2–90.8)	3	75.0 (21.9–98.7)
Calabria	10	4	40.0 (13.7–72.6)	1	10.0 (0.5–45.9)	7	70.0 (35.4–91.9)	6	60.0 (27.4–86.3)
Sicilia	223	84	37.7 (31.4–44.4)	1	0.4 (0.0–2.9)	172	77.1 (70.9–82.4)	92	41.3 (34.8–48.0)
Sardegna	13	9	69.2 (38.9–89.6)	2	15.4 (2.7–46.3)	4	30.8 (10.4–61.1)	9	69.2 (38.9–89.6)

95% CI: 95% Confidence Interval; DSId: Distinct Sequence Identifier; NA: Not Applicable.

Proportions' 95% CIs were computed according to the Wilson method [25].

Supplementary Figure S1. Identification of D8-5963 measles variant in Italian Regions, October 2023-March 2025 (n = 97).

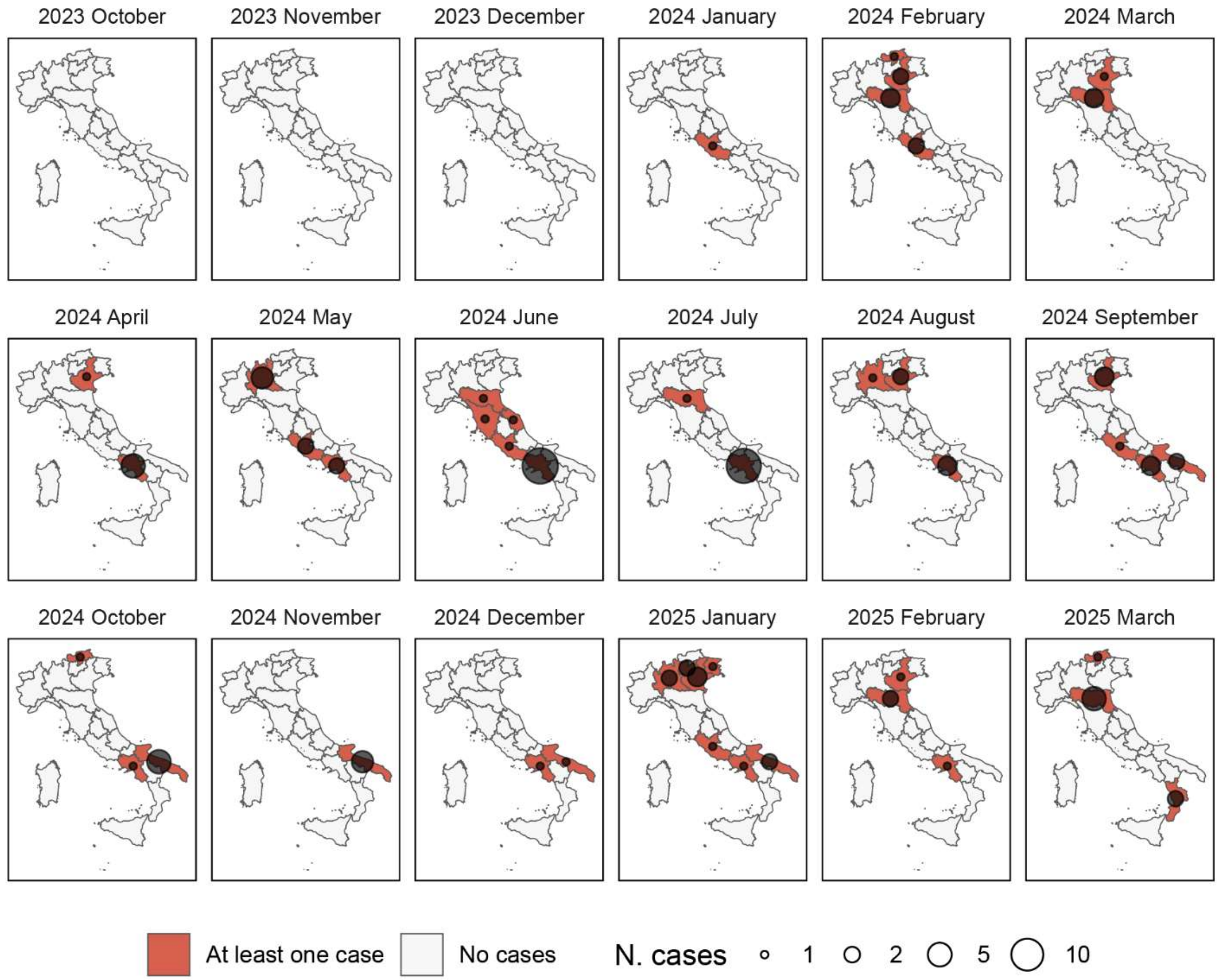

The size of points is proportional to the number of measles cases in a month.

Supplementary Figure S2. Measles identified variants in Italian Regions, by month, October 2023-March 2025 (n = 1,311).

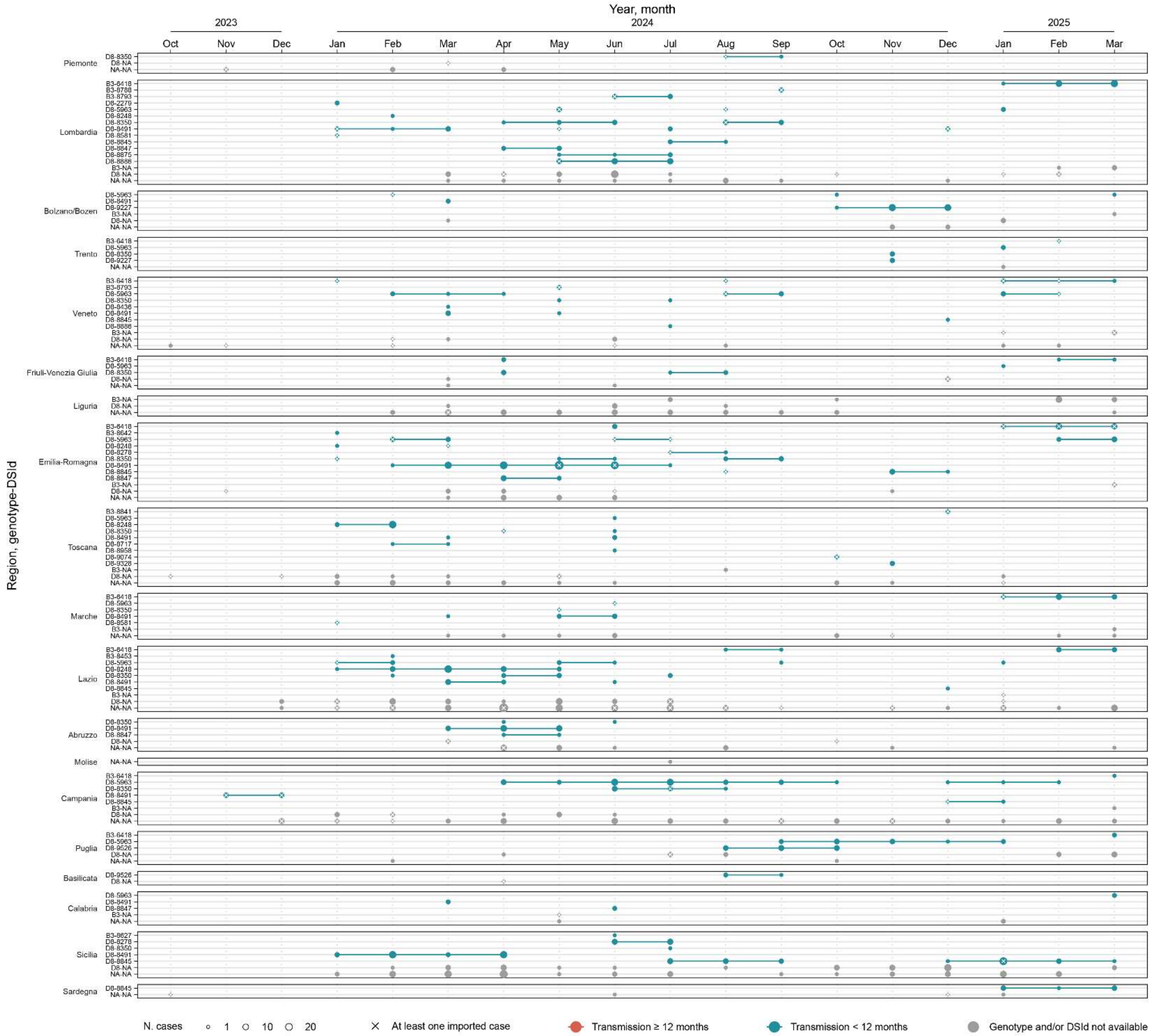

NA: Not available.

The size of points is proportional to the number of measles cases in a month. × denote months with at least one new imported case.
